# Substituent-Guided Cluster Nuclearity for Tetranuclear Iron(III) Compounds with Flat {Fe_4_(μ_3_-O)_2_} Butterfly Core

**DOI:** 10.3390/ijms24065808

**Published:** 2023-03-18

**Authors:** Lorenzo Marchi, Stefano Carlino, Carlo Castellano, Francesco Demartin, Alessandra Forni, Anna M. Ferretti, Alessandro Ponti, Alessandro Pasini, Luca Rigamonti

**Affiliations:** 1 Dipartimento di Scienze Chimiche e Geologiche, Università degli Studi di Modena e Reggio Emilia, Via G. Campi 103, 41125 Modena, Italy; lorenzo.marchi@unimore.it; 2 Dipartimento di Chimica, Università degli Studi di Milano, Via C. Golgi 19, 20133 Milano, Italy; stefano04.85@gmail.com (S.C.); carlo.castellano@unimi.it (C.C.); francesco.demartin@unimi.it (F.D.); alessandro.pasini@unimi.it (A.P.); 3 Istituto di Scienze e Tecnologie Chimiche “Giulio Natta”, Consiglio Nazionale delle Ricerche (SCITEC-CNR), Via C. Golgi 19, 20133 Milano, Italy; alessandra.forni@scitec.cnr.it; 4 Istituto di Scienze e Tecnologie Chimiche “Giulio Natta”, Consiglio Nazionale delle Ricerche (SCITEC-CNR), Via G. Fantoli 16/15, 20138 Milano, Italy; anna.ferretti@scitec.cnr.it (A.M.F.); alessandro.ponti@scitec.cnr.it (A.P.)

**Keywords:** tetradentate ligands, Schiff bases, oligonuclear complexes, X-ray structures, magnetic properties, DFT calculations, halogen bond

## Abstract

The tetranuclear iron(III) compounds [Fe_4_(μ_3_-O)_2_(μ-L^Z^)_4_] (**1**–**3**) were obtained by reaction of FeCl_3_ with the shortened salen-type N_2_O_2_ tetradentate Schiff bases *N,N’*-*bis*(salicylidene)-*o*-Z-phenylmethanediamine H_2_L^Z^ (Z = NO_2_, Cl and OMe, respectively), where the one-carbon bridge between the two iminic nitrogen donor atoms guide preferentially to the formation of oligonuclear species, and the *ortho* position of the substituent Z on the central phenyl ring selectively drives towards Fe_4_ bis-oxido clusters. All compounds show a flat almost-symmetric butterfly-like conformation of the {Fe_4_(μ_3_-O)_2_} core, surrounded by the four Schiff base ligands, as depicted by both the X-ray molecular structures of **1** and **2** and the optimized geometries of all derivatives as obtained by UM06/6-311G(d) DFT calculations. The strength of the antiferromagnetic exchange coupling constants between the iron(III) ions varies among the three derivatives, despite their magnetic cores remain structurally almost unvaried, as well as the coordination of the metal ions, with a distorted octahedral environment for the two-body iron ions, Fe_b_, and a pentacoordination with trigonal bipyramidal geometry for the two-wing iron ions, Fe_w_. The different magnetic behavior within the series of examined compounds may be ascribed to the influence of the electronic features of Z on the electron density distribution (EDD) of the central {Fe_4_(μ_3_-O)_2_} core, substantiated by a Quantum Theory of Atoms In Molecules (QTAIM) topological analysis of the EDD, as obtained by UM06 calculations **1**–**3**.

## 1. Introduction

The need for miniaturized devices for technological applications is driving scientific research into the replacing of existing materials with molecular species of nanometer sizes [1,2,3,4]. Polynuclear compounds of transition metal ions are very promising materials for this purpose because of their tunable electronic and magnetic properties [5,6,7,8], achieved through the modulation of the magnetic exchange interactions. In the search for this variability, until a few years ago, the serendipitous assembly approach led to a huge increase in the amount of synthesized polynuclear complexes with different bridging ligands [9], but a rational approach is most desirable, with precise control of the obtained compounds and their properties by the modification of the ligands [10] and the synthetic conditions [11,12,13].

Schiff bases are well-known ligands that have been used for decades in the synthesis of metal complexes for applications in different fields, such as catalysis [14], new materials and optics [15,16,17]. From the most famous N_2_O_2_ tetradentate Schiff base H_2_salen, formed by the condensation of salicylaldehyde (salH) with ethylenediamine (en) [18,19,20], new functionalized families of structurally modified molecules have been designed for the purpose of obtaining new polynuclear complexes with desired structural features and functionalities [21,22,23]. In particular, the substitution of en with the shorter methylenediamine in condensation with salH yields ligands (called H_2_salben’s when the methylene bridge between the two nitrogen atoms carries a phenyl ring) that preferentially produce oligonuclear compounds by complexation [24,25,26,27].

**Scheme 1:**
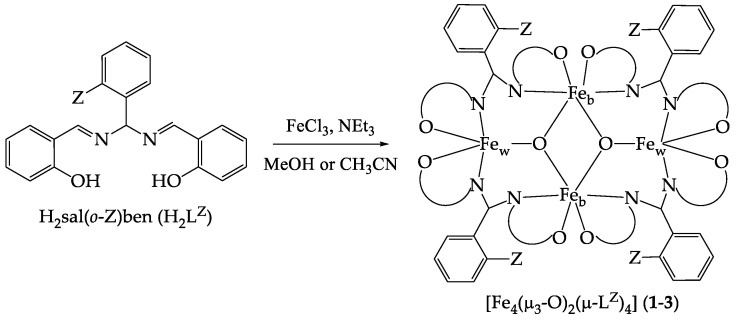
Synthetic scheme of **1**–**3** (Z = NO_2_, Cl, OMe, respectively) and drawing of the arrangement of the Schiff base ligands around the {Fe_4_(μ_3_-O)_2_} butterfly core (sal moieties are sketched as ∩ for clarity; iron ions are divided into those of two body, Fe_b_, and two wing, Fe_w_, ions, as applicable in butterfly-like Fe_4_ clusters).

Based on our previous experience in working with Schiff bases and the preferred isolation of oligonuclear metal clusters [8,24,25,26,27,28,29,30,31], in this work, we decided to exploit the metal complexation ability toward iron(III) of three H_2_sal(*o*-Z)ben derivatives bearing a substituent Z in the *ortho* position of the central phenyl ring (*N,N*’-*bis*(salicylidene)-*o*-Z-phenylmethanediamine) [24,32] (abbreviated as H_2_L^Z^ in this paper) with Z going from NO_2_ to Cl and OMe (Scheme 1). The tetranuclear iron(III) compounds [Fe_4_(μ_3_-O)_2_(μ-L^Z^)_4_] (**1**–**3**, with Z = NO_2_, Cl, OMe, respectively, see Scheme 1) could be selectively obtained by reaction with FeCl_3_ in contrast with the formation of dinuclear species [Fe_2_((μ-OMe)_2_(μ-sal(*p*-Y)ben)_2_] when ligands with substituents Y in *para* position or absent were employed [27]. Here, we report the synthesis and the structural and magnetic characterization of these complexes, revealing the modulation of Z on the exchange coupling constants between the iron ions within the three derivatives. The topological analysis of the computed electron density distribution (EDD) of **1**–**3** will help to discuss the electronic effect of Z.

## 2. Results and Discussion

### 2.1. Synthesis and X-ray Crystal Structures

The reaction of H_2_L^Z^ with FeCl_3_ in non-anhydrous MeOH or MeCN in the presence of NEt_3_ as a base gave the tetranuclear complexes **1**–**3** good yields (see Scheme 1), with the best ones in MeOH. The polynucleation ability of the salben ligands due to the one-carbon bridge between the two iminic nitrogen atoms is clearly confirmed here [24,25,26,27]. There is also the control on the nature of the oligonuclear compound upon substituent shift, since the dinuclear species [Fe_2_((μ-OMe)_2_(μ-sal(*p*-Y)ben)_2_] are isolated when ligands with substituents Y in *para* position or absent were employed [27]. The tetranuclear nature of **1**–**3** is proven by the ESI^+^ mass spectra with the [M+Na]^+^ and [M+1]^+^ peaks and the fragmentation of the [Fe_3_(O)(L^Z^)_3_]^+^ ions (see Experimental Section). Furthermore, single crystals suitable for the X-ray structure determination of **1**·1.5*i*Pr_2_O and **2**·2*i*Pr_2_O were obtained, and the crystal structures could be refined.

**Figure 1:**
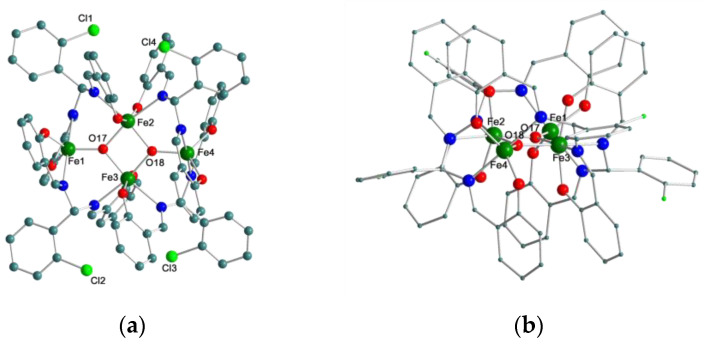
(**a**) Molecular structure of [Fe_4_(μ_3_-O)_2_(μ-L^Cl^)_4_] (**2**) (Fe = dark green, O = red, N = blue, Cl = light green, C = grey; hydrogen atoms omitted for clarity). Fe1 and Fe4 are the Fe_w_ ions, while Fe2 and Fe3 are the Fe_b_ ions. (**b**) View of the tetranuclear cluster **2** almost along the Fe_4_O_2_ plane, highlighting the flat core.

The asymmetric unit of **1**·1.5*i*Pr_2_O contains two independent clusters [Fe_4_(μ_3_-O)_2_(μ-L^NO2^)_4_], A and B, structurally very similar (see Figure S1 for molecule A and an overlap of A and B in Figure S2a in Supplementary Materials), and three *i*Pr_2_O solvent molecules, clathrated in the lattice voids. For **2**·2*i*Pr_2_O, in contrast, only one independent molecule of [Fe_4_(μ_3_-O)_2_(μ-L^Cl^)_4_] and two *i*Pr_2_O molecules are present in the asymmetric unit (Figure 1). Both molecules of **1** and **2** show four iron(III) ions linked together by two triply-bridging oxido anions, giving the {Fe_4_(μ_3_-O)_2_} core. The four dianionic tetradentate ligands sal(*o*-Z)ben^2–^ surround the core, bridging two iron ions each, as shown in Scheme 1 and Figure 1 and Figure S1 in Supplementary Materials.

In such clusters, the {Fe_4_(μ_3_-O)_2_} core adopts a so-called butterfly-like conformation where the iron(III) ions are usually divided into two ‘body’, Fe_b_ (Fe2, Fe3), and two ‘wing’, Fe_w_ (Fe1, Fe4), metal ions [33]. In this butterfly-like conformation, the Fe2–Fe3 fragment features the body of the butterfly, and the Fe1Fe2Fe3 and Fe2Fe3Fe4 triangles schematize the wings, with the Fe1 and Fe4 occupying the tip positions. The Fe_4_O_2_ moieties are essentially planar, with maximum displacements from the least square (l.s.) planes of only 0.082 (Fe4A) and 0.064 Å (Fe4B) for **1**, and 0.027 Å (O17) for **2** (see Figure 1 and Figure S1 in Supplementary Materials). Another proof of the planarity of the metal cores can be detected through the dihedral angles between the l.s. planes defined by the two wings, which are 176.9 and 176.2° (molecules A and B, respectively) in **1** and 179.1° in **2**, very close to the ideal value of 180°. There are few other cases that have been reported in which the two wings lie in the same plane [34,35,36,37], but they are usually associated with higher displacement values of the oxido ions from the Fe_3_ planes, which is not the case here.

The μ_3_-O bridges in **1** are asymmetric: the Fe_w_–O distances (average 1.790 Å) are shorter than the Fe_b_–O ones (average 1.960 Å). Similar asymmetry is also present in **2,** with a short Fe_w_–O average distance of 1.802 Å and a long Fe_b_–O average distance of 1.945 Å (see Table S1 in Supplementary Materials). This feature is in line with values reported in the literature for butterfly-like Fe_4_O_2_ clusters [33], in which the Fe_w_–O distances of our compounds are among the shortest observed. The μ_3_-O bridge asymmetry is evident also by analyzing the Fe–O–Fe angles, ranging from 130.0(3)° to 133.2(3)° in **1** and from 127.81(6)° to 132.71(6)° in **2** for Fe_w_–O–Fe_b_, while the values are much smaller for Fe_b_–O–Fe_b_ (from 96.7(2)° to 97.2(2)° in **1** and 99.31(5)° and 99.64(6)° in **2** (see Table S1 in Supplementary Materials for all values). The opening of the Fe_b_–O–Fe_b_ angles in **2** leads to a slightly longer Fe_b_···Fe_b_ distance (2.9683(4) Å) compared to **1** (2.929(2) and 2.937(2) Å) and to an asymmetry in the Fe_w_ position that brings Fe1 and Fe4 closer to Fe3, compared to Fe2 in **2** (Fe1···Fe2 = 3.4241(4) Å, Fe1···Fe3 = 3.3738(5) Å, and Fe4···Fe2 = 3.4211(5) Å, Fe4···Fe3 = 3.3737(4) Å), while all the Fe_w_···Fe_b_ distances range around 3.41 Å in **1**.

Considering the metal coordination, the two Fe_w_ ions have a trigonal bipyramidal (*tb*) coordination (see Figure 1 and Figure S1 in Supplementary Materials), while usually they show an octahedral (*oh*) geometry [33,34,35,36,37,38,39,40,41,42,43,44,45,46,47,48,49,50,51]; this feature can be ascribed to the ‘strain’ induced by the Schiff base ligands. Considering the trigonal plane formed by the three oxygen atoms coordinated to Fe_w_, the *tb* polyhedra are rotated on average by 70° (**1**) and 84° (**2**) with respect to each other and by about 55° (**1**) and 48° (**2**) with respect to the Fe_4_O_2_ l.s. plane. The two Fe_b_ ions have instead quite a distorted octahedral coordination, still due to the ‘strained’ surrounding ligands (see Table S1 in Supplementary Materials).

The asymmetry in the wing iron ion positions and the different rotation of the *tb* polyhedra can probably be explained by crystal packing and/or the steric and electronic effects of the Z substituents. In particular, the arrangement of the four sal(*o*-Cl)ben^2–^ ligands around the {Fe_4_(μ_3_-O)_2_} core in **2** is noteworthy, where each of the chlorine atoms points towards the center of the adjacent phenyl ring, giving rise to a C–Cl···π halogen bond [52] (average distance of Cl from the l.s. plane through the phenyl ring = 3.66 Å, see Figure 1), due to the interaction between the positive region of the electrostatic potential on the halogen along the extension of the C–Cl bond with the π system of the phenyl ring [53,54,55]. On the other hand, the nitro group in **1** is unable to produce a stabilizing interaction with the π system of the adjacent phenyl ring, determining a more irregular arrangement of the four sal(o-NO_2_)ben^2–^ ligands with respect to the {Fe_4_(μ_3_-O)_2_} core (see Figure S1 in Supplementary Materials). This feature also leads to slightly different conformations of the surrounding ligands, mainly affecting the central phenyl ring carrying the substituent Z, as is clearly perceivable by superimposing the two structures (see Figure S2b in Supplementary Materials for the overlap of **1**A and **2**).

The Fe–O bonds involving the phenoxido oxygen atoms of the Schiff base ligands are in the range 1.865(5)–1.964(5) Å for **1** and 1.8660(12)–1.9698(13) Å for **2**, with usually shorter distances when the wing Fe1 and Fe4 ions are involved. Fe–N bonds are longer than Fe–O ones, ranging from 2.160(6) to 2.234(6) Å in **1** and from 2.1509(15) to 2.2730(13) Å in **2**, but they show the same trend with respect to Fe_w_ and Fe_b_. It is instead hard to discern a direct effect of the substituent Z on the coordination distances.

### 2.2. Optimized Geometries

Aimed at confirming the stability of **3** and ascertaining the influence of Z, if any, on the coordination distances of the iron ions, UM06/6-311G(d) calculations were performed on **1**–**3**. Selected distances are reported in Table 1 in direct comparison with the corresponding experimental ones. The resulting optimized structures are essentially symmetric, unlike the experimental ones, which show a slight degree of asymmetry as described above, probably due to crystal packing effects. The comparison of the Fe_w_–O and Fe_b_–O computed distances with the corresponding experimental average values in **1** and **2** indicates an overall good agreement, with just a little overestimate for the former (1.818 and 1.825 Å vs. 1.790(5) and 1.802(11) Å, respectively) and a better correspondence for the latter (1.951 and 1.947 Å vs. 1.960(5) and 1.945(11) Å, respectively (see Figure S2c,d in Supplementary Materials for an overlap of the optimized and experimental structures).

The computed structures evidence a minor influence of the Z groups on the coordination bond lengths of the central {Fe_4_(μ_3_-O)_2_} cores by comparing the corresponding bond lengths in **1**, **2** and **3**, with the Fe_w_–O and Fe_b_–O distances slightly increasing (1.818, 1.825, 1.829 Å) and decreasing (1.951, 1.947, 1.944 Å), respectively, going from Z = NO_2_ to Cl and then to OMe. The remaining Fe–O distances do not show significant variations along the same series (Fe_w_–O is essentially unvaried, Fe_b_–O slightly increases from 1.956 to 1.960 and 1.967 Å), while more significant, though still small, variations are observed in the Fe–N distances, which decrease for both Fe_w_ (2.170, 2.157, 2.150 Å) and Fe_b_ (2.225, 2.224, 2.211 Å) ions when going from **1** to **3**. The geometry of the {Fe_4_(μ_3_-O)_2_} core is only slightly different in the three complexes, with a lengthening of the Fe_w_···Fe_w_ distance up to 0.028 Å and shortening of the Fe_b_···Fe_b_ one equal to 0.023 Å going from the electron-withdrawing NO_2_ group to the electron-donating OMe group. As already evidenced in previous studies on copper(II) complexes of salen analogues with push–pull structures [15], NO_2_ exerts the strongest electronic effect on the coordination geometry with respect to OMe.

It is worth noting that the calculations correctly reproduce the Cl···π interaction observed in the crystal structure, providing Cl distances from the ring centroids equal to 3.668 and 3.660 Å. This agreement can be ascribed to the ability of the M06 functional to describe weak dispersion-dominated interactions, unlike the B3LYP functional, which has been shown to be unable to model the C–X···π halogen bond [53,54,55]. According to previous CCSD(T) calculations at the complete basis set limit on model systems constituted by benzene and DCl dimers (D = H, HCC, F and NC) in a T-shaped configuration [54], the energy associated with this interaction ranges from –1.43 to –3.38 kcal mol^–1^ at the respective equilibrium distances (from 3.45 to 3.25 Å), according to the electron-withdrawing ability of the D group. It is then expected that the C–Cl···π interaction in **2**, while present, is however quite weak, owing to the observed large Cl···π distance and the non-optimal T-shaped arrangement of the interacting species.

By analyzing the conformation of the ligands in the optimized geometries of **1**–**3** (see Figure S2e in Supplementary Materials), it is apparent the different orientation of the central phenyl ring carries the substituent Z, similar to the experimental structures, while the cores and the coordination environments of the four iron ions are almost superimposed. Moreover, the computed reciprocal rotations of the two *tb* polyhedra with respect to each other (78.5, 81.1 and 83.5° for **1**, **2** and **3**, respectively) and with respect to the Fe_4_O_2_ plane (50.8, 49.5 and 48.3° for **1**, **2** and **3**, respectively) show a lower variability with respect to the X-ray crystal structures but follow the same experimental trend. This indicates that, besides crystal packing effects, the intrinsic electronic and steric effects induced by Z are also responsible for the observed conformational differences between **1** and **2** in the solid state.

### 2.3. Magnetic Properties

**Figure 2:**
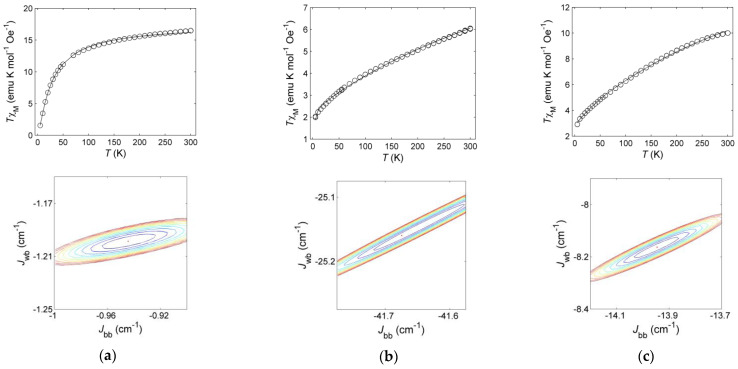
Temperature dependence of the *χ*_M_*T* product of (**a**) **1**, (**b**) **2** and (**c**) **3** (*χ*_M_ is the molar susceptibility referred to an Fe_4_ unit). Top panels: experimental data (circles) and best-fit curves (solid line) based on the simplified butterfly Hamiltonian Equation (1). Bottom panels: constant *ssr* contours from Δ*ssr* = 1 (indigo inner curve) to Δ*ssr* = 10 (brown outer curve); the red dot marks the best-fit point.

The magnetic susceptibility of **1**–**3** was measured between 5 and 300 K. The product *χ*_M_*T* (*χ*_M_ is the molar susceptibility referred to an Fe_4_ unit) of **1** (Figure 2a) is 16.5 ± 0.3 emu K mol^–1^ Oe^–1^ at 300 K, which is slightly lower than that for four uncoupled iron(III) ions (17.5 emu K mol^–1^ Oe^–1^, *g* = 2.00). On cooling, *χ*_M_*T* of **1** decreases slowly down to 50 K and more rapidly at lower temperature up until it reaches the value 1.55 ± 0.03 emu K mol^–1^ Oe^–1^ at 5 K. Such behavior suggests that the iron(III) ions are antiferromagnetically coupled and that the coupling is weak. 

The product *χ*_M_*T* of **2** and **3** (Figure 2b,c) at 300 K is 6.04 ± 0.02 and 10.0 ± 0.2 emu K mol^–1^ Oe^–1^, respectively, which is much lower than that of four uncoupled Fe^3+^ ions. For both **2** and **3**, *χ*_M_*T* decreases upon cooling but, differently from **1**, the decrease is much less curved between 300 and 5 K. Such results suggest that the antiferromagnetic interactions between the iron ions are stronger in **2** and **3** than in **1**, a conclusion seemingly contrasting with the larger *χ*_M_*T* values at low temperature (2.00 ± 0.01 and 2.91 ± 0.06 emu K mol^–1^ Oe^–1^ at 5 K for **2** and **3**, respectively). 

**Scheme 2:**
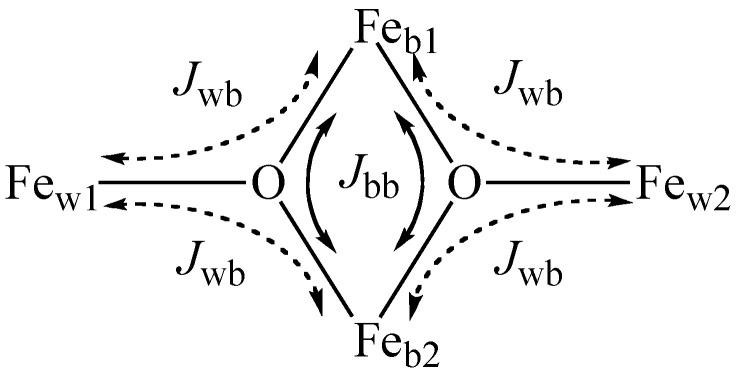
Magnetic exchange pathways in the simplified butterfly Fe_4_ spin system considered during the fitting of the experimental data (*J*_ww_ = 0, see text).

Several super-exchange pathways magnetically connect the wing and body iron ions of the planar {Fe_4_(μ_3_-O)_2_} core in **1**–**3** (see Scheme 2). Thanks to the symmetry of the cores, all wing–wing super-exchange interactions can be considered equal, and the same assumption can be made for wing–body and body–body interactions. Besides, any magnetic communication through the Schiff base ligands can be neglected because of the longer exchange pathway. Within these approximations, the spin system of **1**–**3** can be modelled as a butterfly spin system [56] where the exchange couplings are fully determined by the three coupling constants *J*_bb_ (body–body), *J*_wb_ (wing–body), and *J*_ww_ (wing–wing). In complexes **1**–**3**, the wing–wing exchange can be neglected with respect to the wing–body and body–body interactions because of the longer exchange pathway. By this further assumption, we can set *J*_ww_ = 0 and obtain a simplified butterfly spin system described by the Hamiltonian:

*Ĥ* = –2*J*_bb_ **Ŝ**_b1_**·Ŝ**_b2_ − 2*J*_wb_ (**Ŝ**_w1_**·Ŝ**_b1_ + **Ŝ**_w1_**·Ŝ**_b2_ + **Ŝ**_w2_**·Ŝ**_b1_ + **Ŝ**_w2_**·Ŝ**_b2_) (1)

This Hamiltonian can be treated by the Kambe vector coupling method [57], and the energy of the spin states can be expressed as

*E*(|*S*_bb_, *S*_ww_, *S*〉) = –*J*_bb_ [*S*_bb_(*S*_bb_ + 1) − 35/2] − *J*_wb_ [*S*(*S* + 1) − *S*_bb_(*S*_bb_ + 1) − *S*_ww_(*S*_ww_ + 1)] (2)

where the spin state |*S*_bb_, *S*_ww_, *S*〉 is labelled as to the two intermediate (**Ŝ**_bb_ = **Ŝ**_b1_ + **Ŝ**_b2_ and **Ŝ**_ww_ = **Ŝ**_w1_ + **Ŝ**_w2_) and the total (**Ŝ** = **Ŝ**_bb_ + **Ŝ**_ww_) spin operators. Molar susceptibility *χ*_M_ was calculated by inserting these spin state energies into the Van Vleck equation [56] and was then fitted to the experimental susceptibility data by varying *J*_bb_ and *J*_wb_. In all cases, the *g*-value was fixed to 2.00, as appropriate for high spin iron(III). This model could be successfully applied to the experimental data of **1**–**3** (Figure 2), confirming the starting assumptions.

Before discussing the best-fit results, we need to examine their reliability since the *J*_bb_ values obtained from the magnetic susceptibility data of butterfly-like {Fe_4_(μ_3_-O)_2_} complexes can be largely under-determined [37,43]. This occurs because at low temperature, only |5,5,*S*〉 states are significantly populated, which differ in energy by

Δ*E*(*S*’, *S*) = −*J*_wb_ [*S*’(*S*’ + 1) − *S*(*S* + 1)] (3)

Thus, a change in *J*_bb_ does not affect the energy of the low-lying states and, hence, the magnetic susceptibility *χ*_M_ is scarcely sensitive to *J*_bb_ [35]. To analyze the confidence level of the best-fit parameters, we computed the sum of squared weighted residuals (*ssr*) for a region of the (*J*_bb_, *J*_wb_) plane about the best-fit values and found the constant-value contours of the difference Δ*ssr* = *ssr* − *ssr*_min_, i. e., the deviation from the minimum (best-fit) *ssr* (see Figure 2). The Δ*ssr* contours show a well-defined minimum in the (*J*_bb_, *J*_wb_) plane, proving that the best fit *J*_bb_ and *J*_wb_ values are reliable. Realistic estimates of the parameter errors could be obtained by projecting the appropriate constant Δ*ssr* contours on the *J*_bb_ and *J*_wb_ axes [58], and they are reported in Table 2. Both *J*_bb_ and *J*_wb_ were statistically significant for all the three complexes. The reason for this reliability lies in the peculiar spin level structure of **1**–**3** that we describe below.

Both *J*_wb_ and *J*_bb_ of **1** are weaker than any tetranuclear iron butterfly complex collected in the literature [33], where *J*_wb_ ranges from –91.0 to –37.2 cm^–1^, and *J*_bb_ ranges from –21.8 to –1.2 cm^–1^. Furthermore, *J*_wb_ and *J*_bb_ show comparable strength (see Table 2). The *J*_bb_*/J*_wb_ ratio of **1** is much larger than that of the reported butterfly {Fe_4_(μ_3_-O)_2_} complexes (*J*_bb_*/J*_wb_ ranging from 0.031 to 0.265) [33], with a single exception (*J*_bb_*/J*_wb_ = 1.48) [35]. The ground state of **1** is the |5,5,0〉 singlet state, where the body–body antiferromagnetic interaction is completely frustrated [43]. The energy separation between spin states is small, and many of them are populated even at 5 K (see Figure S3 in Supplementary Materials), including states with *S*_bb_ < 5, because of the weakness of both coupling constants. Thus, *χ*_M_ significantly depends on *J*_bb_ through the energy difference between such states and the |5,5,0〉 ground state (cfr. Equation (3)). That is the reason why *J*_bb_ could be reliably determined from the susceptibility data of **1** [35].

The *J*_bb_ and *J*_wb_ of both **2** and **3** are in line with previously reported values [33] when separately considered, but the body–body exchange interaction is unusually strong compared to the wing–body interaction. Therefore, the body–body exchange interaction is not completely frustrated, resulting in the |3,5,2〉 ground state and the |4,5,1〉 and |2,5,3〉 lowest excited states. Thus, the spin states with unequal *S*_bb_ are populated even at low temperature, again ensuring a reliable determination of *J*_bb_. The *J*_bb_*/J*_wb_ ratio (*ca.* 1.7 for both **2** and **3**) is the highest obtained, and **2** and **3** are among the very few examples of compounds with a {Fe_4_(μ_3_-O)_2_} core possessing a non-singlet ground state (quintet *S* = 2), in addition to the reported complex with *J*_bb_*/J*_wb_ > 1 and *S* = 1 [35]. Different spin ground states can be instead stabilized for Mn_4_O_2_ clusters possessing a butterfly core structure [60,61].

It is not then apparent that a magneto–structural correlation [62] exists between the exchange coupling constants and spin state structure displayed by **1**–**3** and the structural features of their {Fe_4_(μ_3_-O)_2_} cores. The pentacoordination of Fe_w_ for sure introduces a novelty that affects the coupling constants *J*_bb_ and *J*_wb_ and the spin ground state, but this cannot be the discriminating factor, since all derivatives show this feature. The different orientation of the *tb* polyhedra with respect to the Fe_4_O_2_ plane between **1** on one side and **2** and **3** on the other side should not in principle affect the super-exchange magnetic communication path since it mainly depends on Fe–O distances and angles. The latter in any case show a certain non-negligible degree of variability among the three complexes, even if small, that alter the magnetic core. Furthermore, the presence of Cl···π interactions in **2** might further modulate its magnetic features.

### 2.4. Effect of the Substituents and QTAIM Analysis

The main differentiating element in **1**–**3** is for sure the substituent Z on the surrounding ligands, which displays a different electron demand, as shown by the Hammett-like σ*_ortho_* constants [63,64]. Small, similar *J* constants are found when Z is strongly electron-withdrawing (NO_2_, σ*_ortho_* = +0.8), whereas moderately electron-withdrawing (Cl, σ*_ortho_* = +0.2) and electron-donating (OMe, σ*_ortho_* = −0.39) substituents are associated with more negative coupling constants and the peculiar *J*_bb_*/J*_wb_ > 1. It is thus tempting to attribute (at least in part) the different magnetic properties of **1**–**3** to the different electronic availability in the {Fe_4_(μ_3_-O)_2_} core, as we previously observed in electronically modulated Cu_3_(μ_3_-OH) trinuclear copper(II) complexes [8].

We have then performed a QTAIM topological analysis [65] of the computed electron density distribution (EDD) of **1**–**3**, aimed at determining a possible effect of the substituents Z on the EDD features of the metal core [66]. In Table 3 we report the integrated net charges of the iron ions and the μ_3_-oxido anion. The computed charges on the Fe_b_, Fe_w_ and μ_3_-O atoms are not far from the corresponding values obtained from the QTAIM analysis of the experimental EDD for the carboxylate-bridge butterfly-like complex [Fe^III^_4_(μ_3_-O)_2_(O_2_CCMe_3_)_8_(NC_5_H_4_Me)_2_]∙2CH_3_CN [34], amounting to 1.76, 1.67 and –0.91 e, respectively. While very small differences have been obtained along series **1**–**3**, a systematic lowering of the positive charge on Fe_b_ and a concomitant increase in absolute charge on Fe_w_ and μ_3_-O can be detected, resulting in a progressive reduction of the {Fe_4_(μ_3_-O)_2_} positive charge going from the complex with the electron-withdrawing NO_2_ group to the one bearing the electron-donating OMe group. This agrees with a modulating effect produced by Z on the EDD of the central core, which can consequently affect the magnetic properties in our Fe_4_ clusters.

By looking at more detailed information as given by the values of the topological parameters at the Bond Critical Points (BCP) of the central core (see Table 4), we note a rather significant variation in the *ρ*_BCP_ values, and then in the bond strength, of the Fe–O bonds in the three compounds, thus emphasizing the less apparent trend as given by the bond lengths. The strength of the Fe_b_–O bonds increases from **1** to **3**, while that of the Fe_w_–O bonds decreases within the series, to a greater extent with respect to the Fe_b_–O bonds. The ratio of the *ρ*_BCP_ values for the two types of bonds is in fact 0.706 for **1** and 0.742 for **3**. Owing to the large discrepancy between the experimental and theoretical *ρ*_BCP_ values obtained for the Fe–O bonds in the previously reported carboxylate-bridge butterfly-like complex [34], it is quite problematic to draw some conclusion on the different density values obtained for the Fe–O bonds in **1**–**3** with respect to other {Fe_4_(μ_3_-O)_2_} butterfly-like compounds. It is, however, interesting to note that the *ρ*_BCP_ ratios obtained for the two types of bonds in **1**–**3** are significantly larger than the corresponding value previously reported, 0.66 from both experiment and theory [34]. Finally, the character of the Fe–O bonds, as given by the Laplacian ∇^2^*ρ*_BCP_ and the local energy densities *G*_BCP_, *V*_BCP_ and *H*_BCP_ = *G*_BCP_ + *V*_BCP_, is essentially unvaried along the series **1**–**3**, all quantities being indicative of a predominantly closed-shell character for these bonds (∇^2^*ρ*_BCP_ > 0) with quite a small covalence degree, as given by negative but close to zero *H*_BCP_ values.

## 3. Materials and Methods

### 3.1. General

All used chemicals were reagent grade, and solvents were used as received (Sigma Aldrich, Europe). Elemental analyses were performed at the Microanalytical Laboratory at the Università degli Studi di Milano. ESI-MS spectra were recorded on MeOH or MeCN solutions with a LCQ Advantage Thermofluxional instrument. Infrared spectra were recorded as KBr disks using a JASCO FT-IR 410 spectrophotometer with a 2 cm^–1^ resolution. Schiff bases H_2_L^Z^ (Z = NO_2_, Cl, OMe) were obtained following the synthetic method A reported in the literature [32].

### 3.2. Synthesis of [Fe_4_(μ_3_-O)_2_(μ-L^NO2^)_4_] (***1***)

*First method:* FeCl_3_ (0.0651 g, 0.401 mmol) was added to a solution of H_2_L^NO2^ (0.1511 g, 0.4025 mmol) in MeOH (6.0 mL) and NEt_3_ (1.5 mL). The red mixture was left under stirring for 5 h, and then the red solid obtained was filtered, washed with MeOH, *i*Pr_2_O and dried in vacuo (0.1364 g, 77.7%). *Second method:* FeCl_3_ (0.0667 g, 0.411 mmol) was added to a solution of H_2_L^NO2^ (0.1531 g, 0.4079 mmol) in MeCN (9.0 mL) and Et_3_N (1.5 mL) and the red mixture was left under stirring for 5 h. The red solid obtained was filtered, and the reaction mixture was taken to dryness, yielding further solid. Both solids were extensively washed with a MeOH:H_2_O 1:1 mixture and then dried in vacuo (0.1125 g, 63.09%). Anal. Calcd. for C_84_H_60_Fe_4_N_12_O_18_ (1748.82): C, 57.69; H, 3.46; N, 9.61%. Found: C, 57.29; H, 3.65; N, 9.22%. MS (ESI): *m/z* 1771 ([M + Na]^+^, 60%), 1748 ([M + H]^+^, 100), 1302 ([Fe_3_(O)(L^NO2^)_3_]^+^, 95). IR (KBr): 1611 (ν_C=N_), 1319 (ν_NO2_) cm^–1^. Crystals suitable for X-ray diffraction were obtained by diffusion of *i*Pr_2_O into a MeOH solution of the title compound.

### 3.3. Synthesis of [Fe_4_(μ_3_-O)_2_(μ-L^Cl^)_4_] (***2***)

*First method:* The dark-red product was obtained as above in MeOH starting from FeCl_3_ (0.0755 g, 0.465 mmol) and H_2_L^Cl^ (0.1670 g, 0.4578 mmol) (0.1173 g, 60.06%). *Second method:* As above in MeCN from FeCl_3_ (0.1595 g, 0.9833 mmol) and H_2_L^Cl^ (0.3555 g, 0.9744 mmol) (0.1056 g, 25.40%). Anal. Calcd. for C_84_H_60_Cl_4_Fe_4_N_8_O_10_ (1706.64): C, 59.12; H, 3.54; N, 6.57%. Found: C, 59.24; H, 3.50; N, 6.18%. MS (ESI): *m/z* 1728 ([M + Na]^+^, 30%), 1272 ([Fe_3_(O)(L^Cl^)_3_]^+^, 100). IR (KBr): 1611 (ν_C=N_) cm^–1^. Crystals suitable for X-ray diffraction were obtained by diffusion of *i*Pr_2_O into a MeOH solution of the title compound.

### 3.4. Synthesis of [Fe_4_(μ_3_-O)_2_(μ-L^OMe^)_4_] (***3***)

*First method:* The dark-red solid was obtained as above in MeOH from FeCl_3_ (0.1081 g, 0.6665 mmol) and H_2_L^OMe^ (0.2388 g, 0.6626 mmol) (0.1944 g, 69.49%). *Second method:* As above in MeCN from FeCl_3_ (0.1528 g, 0.9420 mmol) and H_2_L^OMe^ (0.3359 g, 0.9320 mmol) (0.2489 g, 63.25%). Anal. Calcd. for C_88_H_72_Fe_4_N_8_O_14_ (1688.97): C, 62.58; H, 4.30; N, 6.76%. Found: C, 62.85; H, 4.31; N, 6.24%. MS (ESI): *m/z* 1711 ([M + Na]^+^, 10%), 1688 ([M + 1]^+^, 15), 1258 ([Fe_3_(O)(L^OMe^)_3_]^+^, 100). IR (KBr): 1613 (ν_C=N_) cm^–1^.

### 3.5. Crystal Structure Determination

**1**·1.5*i*Pr_2_O: C_186_H_162_Fe_8_N_24_O_39_, *M* = 3804.20, monoclinic, *a* = 26.954(5), *b* = 26.974(5), *c* = 25.774(5) Å, *β* = 99.10(1)°, *V* = 18,503(16) Å^3^, *T* = 293(2) K, space group *Cc* (no. 9), Z = 4, μ = (Mo-Kα) 0.690 mm^–1^. A total of 17,490 reflections (9001 unique; *R*_int_ = 0.062) were collected at room temperature, employing a 0.28 × 0.12 × 0.10 mm crystal mounted on a Bruker APEX II CCD diffractometer using graphite-monochromatized Mo-Kα radiation (λ = 0.71073 Å). Final *R*1 [*wR*2] values were 0.0754 [0.2265] on *I* > 2σ(*I*) [all data]. 

**2**·2*i*Pr_2_O: C_96_H_88_Cl_4_Fe_4_N_8_O_12_, *M* = 1910.94, tetragonal, *a* = 25.465(3), *c* = 14.683(2) Å, *V* = 9522(2) Å^3^, *T* = 293(2) K, space group *P–*4 (no. 81), Z = 4, μ = (Mo-Kα) 0.772 mm^–1^. A total of 55,409 reflections (17,810 unique; *R*_int_ = 0.060) were collected as before at room temperature, employing a 0.25 × 0.08 × 0.07 mm crystal. Final *R*1 [*wR*2] values were 0.0585 [0.1693] on *I* > 2σ(*I*) [all data].

Datasets were corrected for Lorentz-polarization effects and for absorption (*SADABS* [67]). The structures were solved by direct methods (*SIR-97* [68]) and completed by iterative cycles of full-matrix least squares refinement on *F*_o_^2^ and Δ*F* synthesis using the *SHELXL-97* [69] program (*WinGX* suite) [70]. Hydrogen atoms, located on the ∆*F* maps, were allowed to ride on their carbon atoms. Crystallographic data for **1**·1.5*i*Pr_2_O and **2**·2*i*Pr_2_O (excluding structure factors) were deposited into the Cambridge Crystallographic Data Centre as supplementary publication no. 883278 and 883279, respectively. These data can be obtained free of charge via www.ccdc.cam.ac.uk/conts/retrieving.html (or from CCDC, 12 Union Road, Cambridge CB2 1EZ, UK; Fax: +44 1223 336033; e-mail: deposit@ccdc.cam.ac.uk).

### 3.6. Theoretical Calculations

Unrestricted DFT calculations were performed on **1**–**3** at the highest spin multiplicity 21 (*S* = 10), assuming all iron(III) ions in their high spin state (*S* = 5/2), using Gaussian 09 [71]. All the structures were optimized *in vacuo* with the 6-311G(d) basis set, starting from the X-ray geometries for **1** (molecule A) and **2**, and using **2** with the appropriated substitutions as a guess starting geometry for **3**. The functionals B3LYP [72,73] and M06 [74] were tested to check their performances in reproducing the X-ray structures of **1** and **2**. The latter functional, which was properly developed to treat organometallic complexes, provided the best agreement with the experimental geometries (see Tables S1 and S2 in Supplementary Materials for a full comparison between computed and experimental bond distances). Quantum Theory of Atoms In Molecules (QTAIM) [65], as implemented in AIMAll [75] was used for evaluating the net charges of the iron and oxido ions.

### 3.7. Magnetic Measurements

The magnetic moment μ of powder samples of **1**–**3** was measured between 5 and 300 K using a Quantum Design MPMS XL-5 SQUID magnetometer. Weighed amounts (about 15 mg) of **1**–**3** were sealed in polycarbonate capsules, and the magnetic moment μ was measured under an applied magnetic field of 1 kOe from 300 K down to 5 K. The molar susceptibility was obtained as *χ*_M_ = (μ/*H*) × (MW/*m*), where MW is the molecular weight of the complex, *m* is the sample mass, and *H* is the applied magnetic field. Diamagnetic contributions were subtracted from μ before calculating *χ*_M_. The ligand diamagnetism was estimated using Pascal’s constants [76].

## 4. Conclusions

The reaction of FeCl_3_ with the Schiff bases H_2_L^Z^ (Z = NO_2_, Cl, OMe) led selectively to the tetranuclear iron compounds **1**–**3**, confirming the oligonucleation ability of these shortened ligands and the control of the nature of the oligonuclear compound upon substituent shift in *ortho* position of the central aryl ring. The analysis of the X-ray molecular structures of **1** and **2** and of the unrestricted-DFT computed geometries of all derivatives shows the similarity of the planar {Fe_4_(μ_3_-O)_2_} cores, together with intramolecular Cl···π halogen bonds in **2**. In contrast, the *J*_bb_ and *J*_wb_ exchange coupling constants in **1**–**3** are different, and the unusually large *J*_bb_/*J*_wb_ ratio for **2** and **3** suggests a quintet (*S* = 2) ground state for these compounds, which add up to another reported example of triplet (*S* = 1) ground state [35] being different from the singlet ground state (*S* = 0) usually reported for butterfly tetrairon(III) complexes [33].

The cores are slightly geometrically affected by the nature of the substituent Z, with the main differentiating factor given by the orientation of the *tb* polyhedra of the Fe_w_ ions with respect to the Fe_4_O_2_ plane. The different spin ground states and the great variability of the *J* values can hardly be attributed to this structural difference. The unequal donating or accepting power of the substituent Z, which changes the EDD at the {Fe_4_(μ_3_-O)_2_} core as confirmed by the topological QTAIM analysis, together with the different conformations of the surrounding ligands due to crystal packing and/or the steric and electronic effects of Z, may contribute to explaining the different magnetic properties of compounds **1**–**3**. Further studies in this respect are certainly necessary and are planned in order to shed more light on our findings. In any case, the latter represents a solid starting point for investigating new butterfly Fe_4_ clusters with different substituents on the surrounding Schiff bases to fully exploit the potentiality of these shortened salen-type ligand molecules as potent oligonucleating and magnetically modulating agents.

## Figures and Tables

**Table 1:** Selected experimental (first row, if available) and UM06/6-311G(d) computed (second row, figures in italics) distances (Å) for **1**, **2** and **3** ^1,2^.

	1·1.5*i*Pr_2_O ^3^	2·2*i*Pr_2_O	3
Fe1–O17	1.784(5), 1.783(5)	1.7994(11)	–
	*1.818*	*1.825*	*1.829*
Fe4–O18	1.797(5), 1.789(5)	1.8040(11)	–
	*1.818*	*1.825*	*1.829*
Fe2–O17	1.948(5), 1.979(5)	1.9380(12)	–
	*1.951*	*1.947*	*1.944*
Fe2–O18	1.967(5), 1.964(5)	1.9461(11)	–
	*1.951*	*1.946*	*1.944*
Fe3–O17	1.970(5), 1.948(5)	1.9567(11)	–
	*1.951*	*1.946*	*1.944*
Fe3–O18	1.939(5), 1.967(5)	1.9389(12)	–
	*1.951*	*1.947*	*1.944*
Fe1–O1	1.865(5), 1.865(5)	1.9021(13)	–
	*1.892*	*1.891*	*1.891*
Fe1–O5	1.925(5), 1.925(5)	1.8662(12)	–
	*1.892*	*1.889*	*1.891*
Fe1–N1	2.160(6), 2.165(7)	2.1636(11)	–
	*2.170*	*2.158*	*2.150*
Fe1–N4	2.193(6), 2.171(6)	2.1930(15)	–
	*2.170*	*2.157*	*2.150*
Fe2–O4	1.964(5), 1.924(5)	1.9698(13)	–
	*1.956*	*1.960*	*1.967*
Fe2–O13	1.954(5), 1.915(6)	1.9319(11)	–
	*1.956*	*1.959*	*1.967*
Fe2–N3	2.211(6), 2.198(7)	2.2609(12)	–
	*2.225*	*2.224*	*2.211*
Fe2–N10	2.207(7), 2.233(6)	2.2729(13)	–
	*2.224*	*2.224*	*2.211*
Fe1···Fe2	3.426(2), 3.427(2)	3.4241(4)	–
	*3.419*	*3.424*	*3.427*
Fe1···Fe3	3.402(2), 3.406(2)	3.3738(5)	–
	*3.419*	*3.426*	*3.427*
Fe4···Fe2	3.413(2), 3.415(2)	3.4210(5)	–
	*3.419*	*3.426*	*3.427*
Fe4···Fe3	3.420(2), 3.428(2)	3.3736(4)	–
	*3.419*	*3.424*	*3.427*
Fe1···Fe4	6.168(2), 6.172(2)	6.1128(7)	–
	*6.155*	*6.174*	*6.183*
Fe2···Fe3	2.929(2), 2.937(2)	2.9683(4)	–
	*2.980*	*2.965*	*2.957*

^1^ O17 and O18 are the triply bridging oxido ions; ^2^ Fe1 and Fe4 are Fe_w_, while Fe2 and Fe3 are Fe_b_; ^3^ the double experimental values are referred to molecules A and B, respectively.

**Table 2:** Best-fit parameters *J*_bb_ and *J*_wb_ (cm^–1^), *J*_bb_*/J*_wb_ ratio, and spin ground state for **1**–**3** obtained by fitting the butterfly model to the experimental *χ*_M_*T* data.

	1 (NO_2_)	2 (Cl) ^1^	3 (OMe)
*J*_bb_ (cm^–1^)	–0.94 ± 0.06	–41.7 ± 0.2	–14.0 ± 0.3
*J*_wb_ (cm^–1^)	–1.20 ± 0.02	–25.2 ± 0.1	–8.2 ± 0.1
*J*_bb_ */J*_wb_	0.79 ± 0.05	1.66 ± 0.01	1.71 ± 0.04
Ground state	|5,5,0〉	|3,5,2〉	|3,5,2〉

^1^ Model augmented with intermolecular exchange, *zj* = –1.05 ± 0.04 cm^–1^ [59].

**Table 3:** Integrated net charge *q* (e) of the atomic basins *Ω* by the QTAIM partitioning in computed EDD of **1**–**3**.

	1 (NO_2_)	2 (Cl)	3 (OMe)
Fe_b_	1.899	1.898	1.897
Fe_w_	1.836	1.838	1.839
μ_3_-O	–1.204	–1.207	–1.208
Σ{Fe_4_(μ_3_-O)_2_}	5.062	5.058	5.056

**Table 4:** Topological properties at selected BCPs of the central core in computed EDD of **1**–**3**.

	*ρ*_BCP_ (e Å^–3^)	∇*^2^ρ*_BCP_ (e Å^–5^)	*G*_BCP_ (Hartree Å^–3^)	*V*_BCP_ (Hartree Å^–3^)	*H*_BCP_ (Hartree Å^–3^)
Compound **1**					
Fe_b_–O	0.628	11.7	0.132	–0.143	–0.011
Fe_w_–O	0.889	16.9	0.210	–0.245	–0.035
Compound **2**					
Fe_b_–O	0.636	11.9	0.134	–0.146	–0.012
Fe_w_–O	0.870	16.6	0.205	–0.237	–0.032
Compound **3**					
Fe_b_–O	0.640	12.0	0.135	–0.147	–0.012
Fe_w_–O	0.863	16.5	0.202	–0.234	–0.032

## Data Availability

The datasets generated during and/or analyzed during the current study are available from the corresponding author on reasonable request.

## Supplementary Materials

The supporting information can be downloaded at: https://www.mdpi.com/article/10.3390/ijms24065808/s1.
