# Supplementary material for: Substituent-Guided Cluster Nuclearity for Tetranuclear Iron(III) Compounds with Flat {Fe4(μ3-O)2} Butterfly Core
Source: Int J Mol Sci. 2023 Mar 18;24(6):5808. doi: 10.3390/ijms24065808 (PMC10057004; doi:10.3390/ijms24065808)
Supplement: Supplementary file 1 [file ijms-24-05808-s001.zip › ijms-2295417-supplementary.pdf]

Substituent-Guided Cluster Nuclearity for Tetranuclear Iron(III) Compounds with Flat $\{Fe_4(\mu_3\text{-}O)_2\}$ Butterfly Core

Lorenzo Marchi, Stefano Carlino, Carlo Castellano, Francesco Demartin, Alessandra Forni, Anna M. Ferretti, Alessandro Ponti, Alessandro Pasini and Luca Rigamonti

SUPPLEMENTARY MATERIALS

Page S2 **Figure S1**. Molecular structure of molecule A of **1**·1.5^{i}Pr$_2$O.

Page S3 **Figure S2**. Overlap among the X-ray molecular structures of **1**A, **1**B and **2**, and the optimized geometries of **1**, **2** and **3**.

Page S4 **Table S1**. Experimental bond distances and angles for compounds **1**·1.5^{i}Pr$_2$O and **2**·2^{i}Pr$_2$O from X-ray molecular structures.

Page S8 **Table S2**. Selected distances for compounds **1**, **2** and **3** computed at the UB3LYP/6-31G(d), UM06/6-31G(d) and UM06/6-311G(d) levels of theory.

Page S10 **Figure S3**. Spin states of compounds **1**–**3** as obtained by the best-fit procedure.

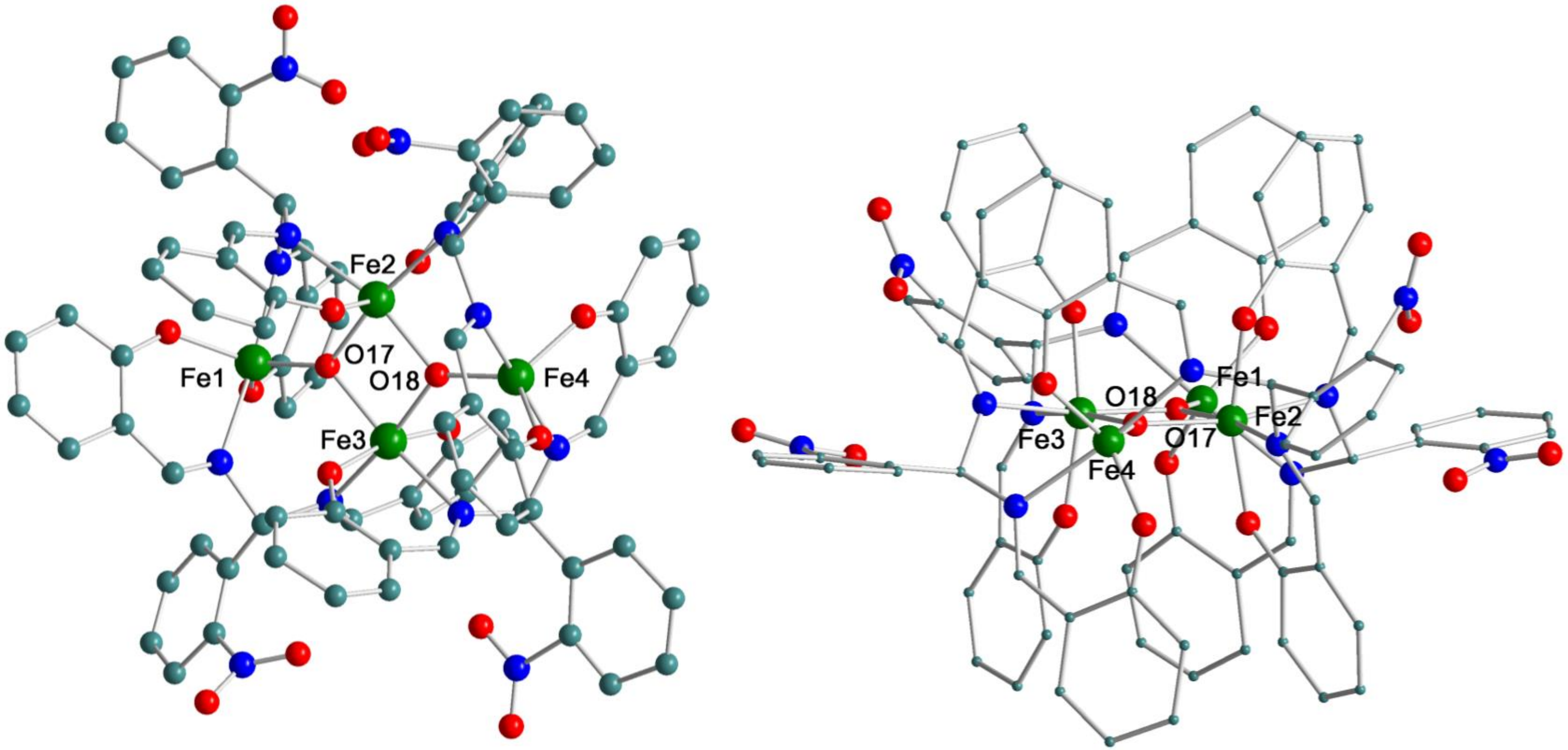

Figure S1. Molecular structure of molecule A of **1**·1.5^{i}Pr$_2$O, in which the additional A symbols are omitted in the main atom numbering (dark green = Fe, red = O, blue = N, grey = C; hydrogen atoms are omitted for clarity).

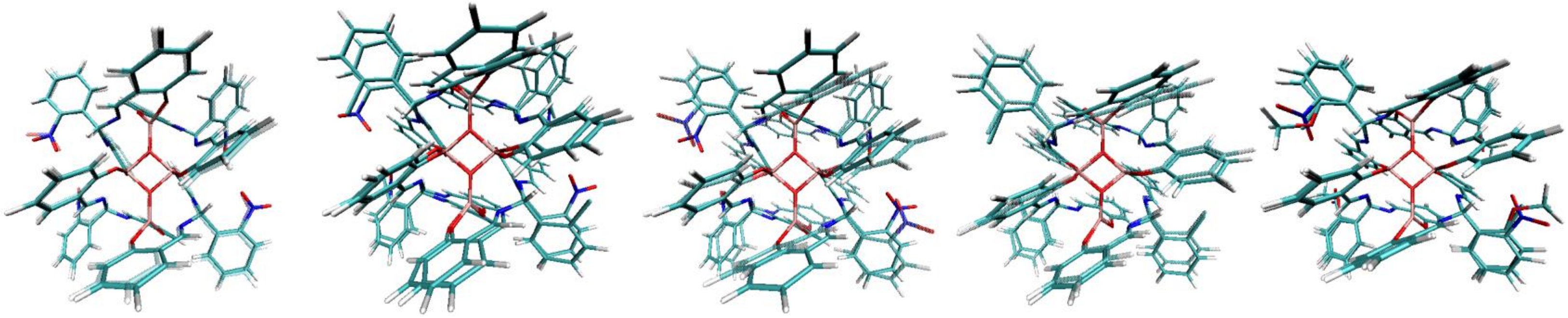

(a) (b) (c) (d) (e)

Figure S2. Overlap of (a) the X-ray molecular structures of **1**A and **1**B (molecule B transparent); (b) the X-ray molecular structures of **1**A and **2**; (c) the X-ray molecular structures of **1**A and the optimized geometry of **1** (the latter transparent); (d) the X-ray molecular structures of **2** and the optimized geometry of **2** (the latter transparent); and (e) the optimized geometries of **1**–**3**. For seek of clarity, Fe–N bonds are not drawn.

Table S1. Experimental bond distances (Å) and angles (°) for compounds **1**·1.5^{i}Pr$_2$O and **2**·2^{i}Pr$_2$O from X-ray molecular structures.

	**1**·1.5^{i}Pr$_2$O		**2**·2^{i}Pr$_2$O
	molecule A	molecule B	
Fe1 distances			
Fe1–N1	2.160(6)	2.165(7)	2.1636(12)
Fe1–N4	2.193(6)	2.171(6)	2.1930(15)
Fe1–O1	1.865(5)	1.865(5)	1.9021(13)
Fe1–O5	1.925(5)	1.925(5)	1.8662(12)
Fe1–O17	1.784(5)	1.783(5)	1.7994(11)
Fe2 distances			
Fe2–N3	2.211(6)	2.198(7)	2.2609(12)
Fe2–N10	2.207(7)	2.233(6)	2.2729(13)
Fe2–O4	1.964(5)	1.924(5)	1.9698(13)
Fe2–O13	1.954(5)	1.915(6)	1.9319(11)
Fe2–O17	1.948(5)	1.979(5)	1.9380(12)
Fe2–O18	1.967(5)	1.964(5)	1.9461(11)
Fe3 distances			
Fe3–N6	2.208(6)	2.214(6)	2.2237(14)
Fe3–N7	2.234(6)	2.226(6)	2.2242(15)
Fe3–O8	1.941(5)	1.961(5)	1.9687(13)
Fe3–O9	1.886(5)	1.954(6)	1.9426(14)
Fe3–O17	1.970(5)	1.948(5)	1.9567(11)
Fe3–O18	1.939(5)	1.967(5)	1.9389(12)
Fe4 distances			
Fe4–N9	2.212(6)	2.181(6)	2.2074(15)

Fe4–N12	2.171(6)	2.206(6)	2.1518(14)
Fe4–O12	1.889(5)	1.879(6)	1.8809(13)
Fe4–O16	1.892(6)	1.900(5)	1.8907(13)
Fe4–O18	1.797(5)	1.789(5)	1.8040(11)
Fe···Fe distances			
Fe1···Fe2	3.426(2)	3.427(2)	3.4241(4)
Fe1···Fe3	3.402(2)	3.406(2)	3.3738(5)
Fe2···Fe3	2.929(2)	2.937(2)	2.9683(4)
Fe2···Fe4	3.413(2)	3.415(2)	3.4211(5)
Fe3···Fe4	3.420(2)	3.428(2)	3.3737(4)
Fe1···Fe4	6.168(2)	6.172(2)	6.1129(7)
μ_3-O angles			
Fe1–O17–Fe2	133.2(3)	131.3(3)	132.71(6)
Fe1–O17–Fe3	130.0(3)	131.8(3)	127.81(6)
Fe2–O17–Fe3	96.8(2)	96.8(2)	99.31(5)
Fe4–O18–Fe2	130.1(3)	131.0(3)	131.60(7)
Fe4–O18–Fe3	132.5(3)	131.7(3)	128.64(6)
Fe2–O18–Fe3	97.2(2)	96.7(2)	99.64(5)
Fe1 angles			
O1–Fe1–O5	113.8(2)	115.4(2)	114.09(6)
O5–Fe1–O17	123.8(2)	124.9(2)	119.60(5)
O17–Fe1–O1	122.4(2)	119.7(2)	126.24(6)
N1–Fe1–O1	89.7(2)	87.0(2)	87.49(5)
N1–Fe1–O5	90.4(2)	91.7(2)	95.34(5)
N1–Fe1–O17	90.5(2)	92.1(2)	90.22(5)

N4–Fe1–O1	89.6(2)	93.6(2)	89.39(5)
N4–Fe1–O5	85.7(2)	83.9(2)	87.12(5)
N4–Fe1–O17	93.7(2)	91.6(2)	90.57(5)
N1–Fe1–N4	175.4(2)	175.4(2)	176.62(5)
Fe2 angles			
O17–Fe2–O18	82.9(2)	82.9(2)	80.59(5)
O18–Fe2–N10	88.6(2)	88.7(2)	87.11(5)
N10–Fe2–N3	104.3(2)	103.8(2)	104.64(5)
N3–Fe2–O17	84.8(2)	85.5(2)	87.87(4)
O13–Fe2–O17	92.7(2)	91.8(2)	102.07(5)
O13–Fe2–O18	100.1(2)	99.5(2)	96.04(5)
O13–Fe2–N10	83.8(2)	83.8(2)	82.97(5)
O13–Fe2–N3	89.2(2)	90.1(2)	82.55(5)
O4–Fe2–O17	99.8(2)	102.5(2)	95.87(5)
O4–Fe2–O18	92.3(2)	90.2(2)	102.57(5)
O4–Fe2–N10	85.5(2)	83.4(2)	82.97(5)
O4–Fe2–N3	81.1(2)	83.3(2)	82.31(5)
O17–Fe2–N10	170.2(2)	169.7(2)	167.10(5)
O18–Fe2–N3	164.9(2)	165.1(2)	167.84(5)
O13–Fe2–O4	163.4(2)	163.6(3)	155.99(5)
Fe3 angles			
O17–Fe3–O18	83.1(2)	83.6(2)	80.31(5)
O18–Fe3–N7	85.1(2)	84.0(2)	89.65(5)
N7–Fe3–N6	103.8(2)	104.3(2)	100.75(5)
N6–Fe3–O17	88.7(2)	88.7(2)	89.33(5)

O8–Fe3–O17	98.1(2)	99.0(2)	94.05(5)
O8–Fe3–O18	90.6(2)	93.3(2)	97.65(5)
O8–Fe3–N6	84.0(2)	82.0(2)	83.92(5)
O8–Fe3–N7	89.6(2)	88.7(2)	84.69(6)
O9–Fe3–O17	90.3(2)	91.4(2)	99.16(6)
O9–Fe3–O18	100.3(2)	99.3(2)	95.58(6)
O9–Fe3–N6	86.2(2)	86.8(2)	85.08(6)
O9–Fe3–N7	84.4(2)	83.8(2)	84.27(6)
O17–Fe3–N7	166.0(2)	165.7(2)	169.63(5)
O18–Fe3–N6	169.5(2)	170.3(2)	169.60(5)
O8–Fe3–O9	167.0(2)	164.6(2)	162.68(6)
Fe4 angles			
O18–Fe4–O12	118.5(2)	122.4(2)	123.22(6)
O12–Fe4–O16	116.0(2)	112.7(2)	115.03(6)
O16–Fe4–O18	125.4(2)	124.8(2)	121.75(6)
N9–Fe4–O18	90.8(2)	93.2(2)	88.92(5)
N9–Fe4–O12	87.9(2)	88.8(2)	85.32(6)
N9–Fe4–O16	89.2(2)	89.1(2)	96.30(6)
N12–Fe4–O18	92.1(2)	91.8(2)	87.44(5)
N12–Fe4–O12	95.2(2)	90.2(2)	94.60(5)
N12–Fe4–O16	85.0(2)	86.4(2)	87.73(6)
N9–Fe4–N12	174.2(2)	174.6(2)	175.60(5)

Table S2. Selected distances (Å) for compounds **1**, **2** and **3** computed at the UB3LYP/6-31G(d) (1st row), UM06/6-31G(d) (2nd row) and UM06/6-311G(d) (3rd row) levels of theory. Experimental average values for **1**·1.5^{i}Pr$_2$O and **2**·2^{i}Pr$_2$O are reported in *italics* on side columns.[1]

	**1**	**1**·1.5^{i}Pr$_2$O	**2**	**2**·2^{i}Pr$_2$O	**3**
Fe1–O17	1.835		1.844		1.846
	1.823		1.828		1.834
	1.818	*1.788*	1.825	*1.802*	1.829
Fe2–O17	1.983		1.984		1.976
	1.952		1.948		1.946
	1.951	*1.960*	1.947	*1.945*	1.944
Fe1–O1	1.912		1.914		1.912
	1.891		1.889		1.892
	1.892	*1.892*	1.890	*1.885*	1.891
Fe1–N1	2.196		2.187		2.177
	2.167		2.158		2.147
	2.170	*2.182*	2.157	*2.178*	2.150
Fe2–O4	1.935		1.939		1.943
	1.948		1.950		1.961
	1.956	*1.937*	1.959	*1.953*	1.967
Fe2–N3	2.313		2.307		2.309
	2.241		2.239		2.224
	2.224	*2.216*	2.224	*2.245*	2.211
Fe1···Fe2	3.463		3.476		3.467
	3.422		3.426		3.431
	3.419	*3.417*	3.425	*3.398*	3.427

Fe1···Fe4	6.229		6.262		6.243
	6.156		6.174		6.187
	6.155	*6.170*	6.174	*6.113*	6.183
Fe2···Fe3	3.030		3.020		3.018
	2.990		2.973		2.967
	2.980	*2.933*	2.965	*2.968*	2.957

[1] for computed geometries, only distances between symmetry-unique atoms are reported, considering that the complexes approximate very close the *D*2 point-group symmetry. The values reported for the experimental geometries (Table S1) are obtained by averaging the distances between equivalent atoms hypothesizing the same *D*2 symmetry. Corresponding experimental distances of molecules A and B of **1**·1.5$^{i}Pr_2O$ are further averaged between them.

Compound **1**	Compound **2**	Compound **3**

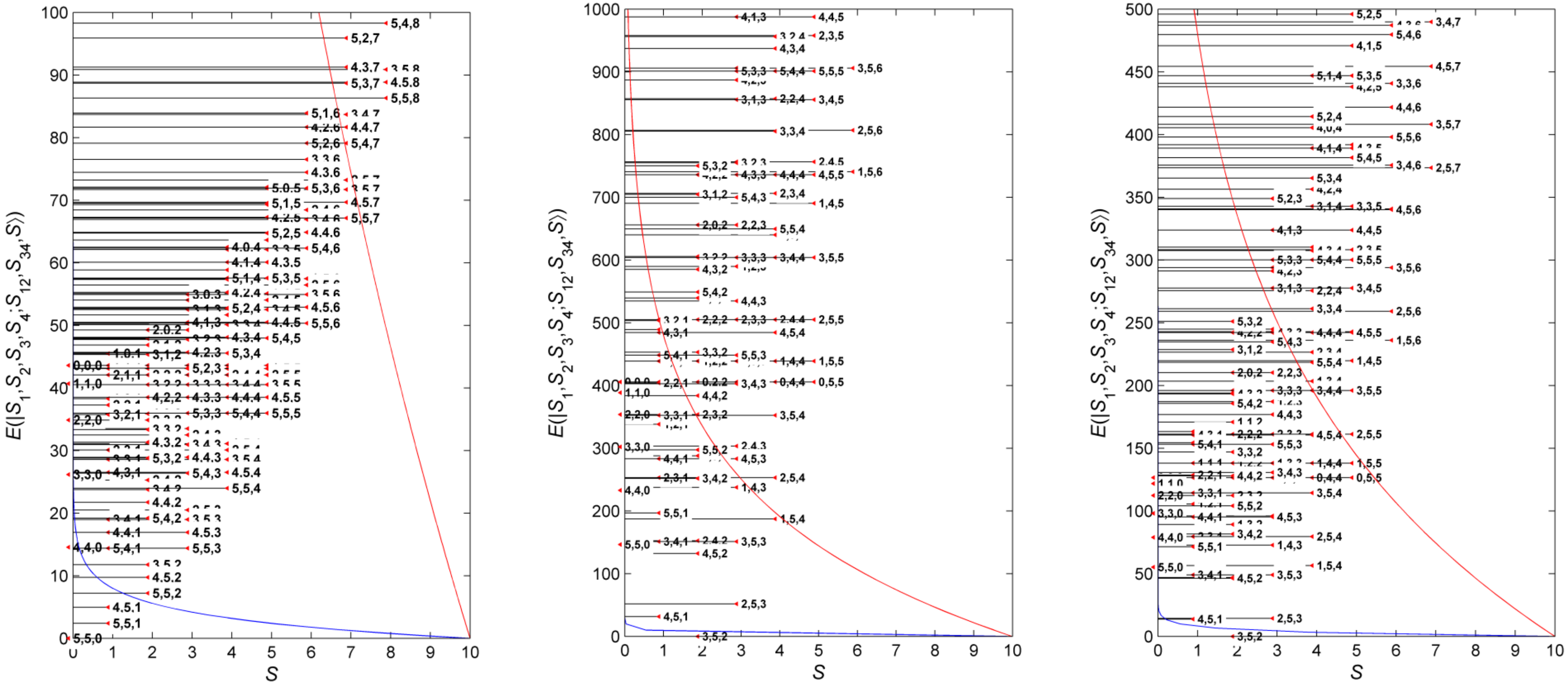

Figure S3. Spin states of compounds **1**–**3** as obtained by the best-fit procedure. The states are ordered as to their energy E (cm^{-1}) from the ground state and labeled as | S_{bb}=S_{12}, S_{ww}=S_{34}, S ⟩. The blue and red lines indicate the Boltzmann population of the spin states at 5 and 300 K, respectively.
